# Development of a FHIR-based Korean IPS Data Pipeline and User-Centered UI Design

**DOI:** 10.1038/s41598-025-33390-z

**Published:** 2026-01-06

**Authors:** Byeonggu Kim, Jisan Lee

**Affiliations:** Department of Nursing, Gangneung-Wonju National University, 150 Namwon-ro, Heungeop-myeon, Wonju, Gangwon State Republic of Korea

**Keywords:** Digital health, Health information interoperability, Data analysis, User-Centered design, Business and industry, Computational biology and bioinformatics, Health care, Medical research, Scientific community

## Abstract

The International Patient Summary (IPS) is a minimal data standard enabling rapid access to essential health information across institutions and borders. Korea provides Fast Healthcare Interoperability Resources (FHIR) data through the “My Health Record” application, which utilizes an implementation guide (IG) inheriting from the KR Core FHIR profiles.  However, a standardized workflow for transforming these domestic FHIR resources into IPS-compliant data has not yet been established.

This study aimed to assess the feasibility of implementing an IPS-compliant patient summary in Korea using existing FHIR-based resources and national profiles. First, a literature review confirmed IPS as a global standard supporting interoperability and patient-centered care. Second, a gap analysis revealed that six of the seven IPS-required and recommended components successfully mapped to ten KR Core profiles. However, the *Device* component and the *MedicationStatement* profile remained unmapped due to the lack of corresponding definitions in the KR Core. Third, real-world FHIR data from three individuals were transformed using ChatGPT-4o into IPS-compatible formats and validated via HAPI FHIR and SMART FRED tools. Fourth, user requirements were identified through personas and expert consultations, highlighting the need for summary and timeline-based UI elements. Fifth, a user interface was developed using Figma based on these requirements.

Overall, approximately 86% of required IPS data elements were represented using existing Korean FHIR-based resources. These findings demonstrate the technical feasibility of IPS implementation in Korea, while also highlighting current gaps in terminology coverage and profile alignment. Future work should focus on multi-site validation, increased automation of mapping processes, and governance frameworks to support scalable and reproducible IPS deployment.

## Introduction

The International Patient Summary (IPS) is a standardized system designed to provide a minimal yet essential set of core medical information, enabling a concise overview of a patient’s health status. It is based on a non-exhaustive set of data elements, including critical items such as current medications and diagnoses^1^. The IPS is not limited to any particular medical specialty or condition and is structured to facilitate rapid access to essential patient information by healthcare providers in sporadic or cross-border clinical contexts^2^. The IPS defines three required components—Problems, Allergies and Intolerances, and Medication Summary (comprising profiles such as MedicationStatement and MedicationRequest)—alongside four recommended components: Immunizations, Results, History of Procedures, and Medical Devices (comprising profiles such as Device and DeviceUseStatement) (Table 1). As a potential solution to challenges posed by information scarcity or overload in clinical settings, the IPS is gaining attention for its capacity to support prompt clinical decision-making, particularly in scenarios where the patient is unconscious or otherwise unable to communicate, by including data on past medical history, underlying conditions, and current medications. The IPS is developed in accordance with the international Health Level 7 (HL7) Fast Healthcare Interoperability Resources (FHIR) standard, and its implementation must adhere to the guidelines set forth in the International Patient Summary Implementation Guide (IPS IG)^3^.

Countries around the world are undertaking various initiatives to leverage the IPS to enhance the integration and accessibility of health information. In the United States, CommonHealth collaborates with over 400 healthcare institutions to enable the sharing of patients’ electronic health records via QR codes^4^, while Wa Verify + in Washington State utilizes the SMART Health Links framework—based on FHIR—to integrate governmental and private-sector medical records into an IPS-compliant format^5^. The European Union ensures continuity of care across member states through the MyHealth@EU platform^6^. In the United Kingdom, the Single Patient Record (SPR) system—launched in 2024 as part of the National Health Service (NHS) electronic health record initiative—consolidates all patient data, enabling access to a Summary Care Record (SCR) that contains only essential medical information^7^.

**Table 1:** IPS components and profiles.

	Components	IPS IG profile
IPS required	Allergies and intolerances	AllergyIntolerance (IPS)
	Medication summary	Medication (IPS)
		MedicationRequest (IPS)
		MedicationStatement (IPS)
	Problems	Condition (IPS)
IPS recommended	Results	DiagnosticReport (IPS)
	History of procedures	Procedure (IPS)
	immunizations	Immunization (IPS)
	Medical devices	Device (IPS)
		Device - performer or observer (IPS)
		DeviceUseStatement (IPS)

Saudi Arabia issues IPS cards to Hajj pilgrims, a measure that contributed to a 40.2% reduction in mortality during the 2023 pilgrimage compared to the previous year^8^. Japan has transitioned its legacy SS-MIX2 system to a FHIR-based architecture and is currently developing a discharge summary system compliant with the FHIR standard^2^. New Zealand has implemented an IPS-based national summary under the name NZIPS (New Zealand International Patient Summary)^10^. In Brazil, a mapping initiative is underway to align the terminology used in the national health data network (RNDS) with international standards, thereby facilitating the integration of IPS into the country’s existing healthcare infrastructure^11^.

To support healthcare data interoperability, numerous countries have developed national-level FHIR implementation guides (IGs). The United States, under the leadership of the Office of the National Coordinator for Health Information Technology (ONC) of the Department of Health and Human Services (HHS), introduced the US Core in 2014^12^. Australia released the AU Base in 2019 via HL7 Australia^13^, while the UK’s NHS launched the UK Core in 2018^14^. Similarly, HL7 Switzerland developed the CH Core^15^ and HL7 Canada published Canadian Baseline^16^.

In Korea, HL7 Korea and the Korea Health Information Service (KHIS) jointly developed the KR Core in 2023. Based on HL7 FHIR R4, the KR Core enhances the interoperability and quality assurance of domestic healthcare data exchange. It incorporates the Korean Core Data for Interoperability (KR CDI) requirements and is formally defined through the KR Core Implementation Guide (KR Core IG). Currently, the KR Core IG comprises a total of 31 profiles, covering key clinical domains essential for IPS alignment, such as AllergyIntolerance, Medication, and Condition. this structure supports efficient and interoperable data utilization across healthcare systems in Korea^17^ (Table 2).

**Table 2:** KR Core profile (relevant with IPS)]

KR Core profiles	KR Core allergyintolerance profile
	KR Core medication profile
	KR Core medicationrequest profile
	KR Core condition profile for chief complaint
	KR Core condition profile for encounter diagnosis
	KR Core diagnosticreport profile for diagnostic imaging
	KR Core diagnosticreport profile for function tests
	KR Core diagnosticreport profile for laboratory results
	KR Core diagnosticreport profile for pathology results
	KR Core procedure profile
	KR Core immunization profile

A representative example of the KR Core is the “My Healthway”, which is accessible through the My Health Record application.While MyHealthway is a primary implementation of the KR Core, it specifically inherits the KR Core profiles and adds further constraints to ensure data consistency within the My Health Record application. This platform enables access to public institution and medical institution data, both of which are shareable in FHIR format^18^. Patient-generated data from hospitals can be disseminated via the Health Information Highway, allowing individuals to manage their personal health records. Although the application supports the sharing of health data in FHIR file format, there remains a lack of mechanisms to extract and concisely summarize this information for effective clinical communication. Therefore, it is essential to develop a Korean adaptation of the IPS using FHIR-compatible data extracted from the My Health Record application.

The objective of this study is to design a FHIR data pipeline and user interface (UI) that integrates user data from the My Health Record application, to implement a Korean version of the IPS.

## Methods

**Fig. 1:**
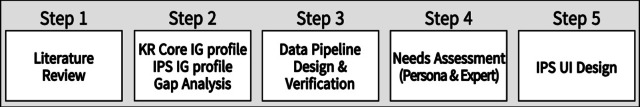
Steps of this study

This study was conducted in a series of structured steps. Step 1 involved a literature review to investigate the global rationale for and implementation strategies of the IPS. In Step 2, a gap analysis was performed between the KR Core Implementation Guide (KR Core IG) profiles and the IPS Implementation Guide (IPS IG) profiles. Based on these findings, Step 3 entailed the design of a Korean IPS data pipeline utilizing FHIR data extracted from the My Health Record application. Subsequently, Step 4 addressed user requirements through the development of personas and usage scenarios, as well as expert consultations. Finally, Step 5 focused on the design of a Korean IPS user interface (UI), informed by the consolidated requirements identified in the previous step (Fig. 1).

### Step 1 literature review

To explore the value, clinical value, and adoption process of the IPS, a comprehensive literature review was conducted. This review encompassed not only academic publications but also gray literature, including reports and presentation materials (e.g., slide decks) released by reputable organizations such as HL7 and the Global Digital Health Partnership (GDHP).

The search was performed using Google Scholar and Google Web Search, based on the following criteria:

- Search terms: (“IPS” OR “International Patient Summary”) AND (“HL7 FHIR” OR “Patient Summary” OR “interoperability”).
- Language: English-language publications.
- Publication period: Within the last 10 years.

The selection of literature was guided by the relevance of content, particularly about the implementation of IPS, its clinical applications, and its association with healthcare data interoperability. Documents were categorized based on predefined inclusion and exclusion criteria.

### Step 2 gap analysis between KR Core IG profiles and IPS IG profiles

As a data-level design step aimed at constructing the system architecture for a Korean implementation of IPS, a gap analysis was conducted to evaluate the practical applicability of IPS. This process involved a comparative examination of the profile elements defined in the KR Core Implementation Guide (KR Core IG) and those specified in the IPS IG.

The seven IPS components—three required and four recommended—were treated as essential for the development of a Korean IPS. Accordingly, each profile element outlined in the official IPS IG documentation^3^ was first mapped to its corresponding IPS Required and Recommended categories. Following this, the mapped IPS IG profiles were further aligned with relevant KR Core profiles, with due consideration of their clinical significance.

This mapping process was jointly conducted by a PhD-level nursing expert and an undergraduate student majoring in nursing. Furthermore, to ensure semantic interoperability, a detailed comparison of profile structures at the resource level was performed. This was necessary because discrepancies in the use of resources or extensions between profiles in FHIR can hinder interoperability. Thus, the profiles defined in the official IPS IG and KR Core IG documents were systematically analyzed and compared at the resource level.

### Step 3. Design of a Korean IPS data pipeline utilizing FHIR data from the “My health Record” application

To assess the practical feasibility of data utilization, FHIR data extractable from the “My Health Record” app was processed and transformed using ChatGPT-4o. This transformation aimed to evaluate whether the extracted data conform to IPS specifications. Based on this analysis, a Korean IPS data pipeline was designed.

#### Step 3.1. Transformation of FHIR data from “My health Record” application into IPS IG format

Among the data elements extractable from the “My Health Record” application, those most closely aligned with the IPS Required and Recommended categories—namely, medication, immunizations, and data from Seoul National University Hospital(SNUH)’s Medical MyHealthData—were extracted in FHIR-compliant .json format. Data were obtained from three researchers participating in this study, with each dataset corresponding to a single day or a single healthcare institution.

A gap analysis was conducted to assess the alignment of the extracted data with the IPS IG profiles, specifically Medication, MedicationStatement, MedicationRequest, Procedure, and Immunization. Two nursing informatics researchers independently carried out the analysis, and results were subsequently compared. The comparison focused on ResourceType, nested resource structures, codes, and code systems.

Following this, data transformation was performed using ChatGPT-4o. Based on the analysis results, specific transformation commands were constructed in prompt format. For the Procedure profile, EDI codes in the generated output were manually replaced with SNOMED CT codes by the researchers using the excisting EDI–SNOMED CT mapping table.

#### Step 3.2. Verification of transformed FHIR data from “My health Record” application

Verification of the transformed data was conducted using a two-tiered approach to ensure both structural correctness and accurate representation.

First, syntactic validation was performed to verify conformity to the FHIR R4 base structure. The datasets were uploaded to the HAPI FHIR Server^19^, a public server endpoint used for open testing. The Create interaction (HTTP POST) was employed, allowing the server to assign resource IDs automatically; consequently, errors related to referential integrity (e.g., unresolved references to non-existent server-side data) were excluded from the scope of this validation.

Second, visual verification was conducted to ensure that the data were correctly parsed and meaningful. The generated data were uploaded to SMART FRED^9^ to verify effective rendering and parsing. This tool displayed the FHIR resources in a human-readable GUI, allowing researchers to visually confirm that the structural elements and manually mapped codes were correctly represented without parsing errors.

#### Step 3.3. Design of the data pipeline

Referencing previous studies on FHIR transformation systems^20,21^, a modular data pipeline was designed to enhance semantic interoperability. Accordingly, an ETL (Extract, Transform, Load) data pipeline was constructed for the validated profiles: Medication, MedicationRequest, Immunization, and Procedure.

### Step 4. Derivation of user requirements

#### Step 4.1. Persona and scenario-based identification of user needs

Before UI design, user needs were identified to determine the core functionalities of the Korean IPS system. This process adopted the MUN (Method for driving User’s Needs) approach within the MASUN (Method of App Selection based on User Needs) framework. The MUN method utilizes brainstorming and mind mapping to gain a deep understanding of the user environment^22^. Based on this, personas and usage scenarios were constructed to concretely answer the question: “What needs would a hypothetical user have when interacting with the system?”

To visualize and analyze user experiences from these scenarios, Customer Journey Mapping (CJM) was employed^23^. CJM visualizes the scenario flow along the horizontal axis and identifies emerging problems or key elements on the vertical axis, enabling the identification of issues at each stage of the user journey. The MUN approach concludes by organizing the identified user needs into a checklist format, which is then refined through expert validation^22^.

To verify the practical relevance of the derived results, validation was conducted by four experts: one representative of a diabetes patient advocacy group (Smart Patient), two medical informatics specialists, and one emergency physician with dual expertise in medical informatics.

#### Step 4.2. Expert consultation-based identification of user needs

In addition to the findings from the literature review, data gap analysis, and persona-based needs assessment, four consultation questions were developed based on queries that arose during the study. These included one question related to UI design, two regarding data, and one concerning data reliability. Each question was designed to include both multiple-choice options and space for subjective input.

Responses were obtained from a panel of experts: two emergency medicine professors, one UX design specialist, one FHIR expert, and one industry professional with experience in developing patient summaries.

### Step 5. UI design

Based on insights from the literature review, data gap analysis, and user requirements elicitation, the UI was designed using Figma.

## Results

### Step 1 literature review

#### Step 1.1. Selected literature

**Fig. 2:**
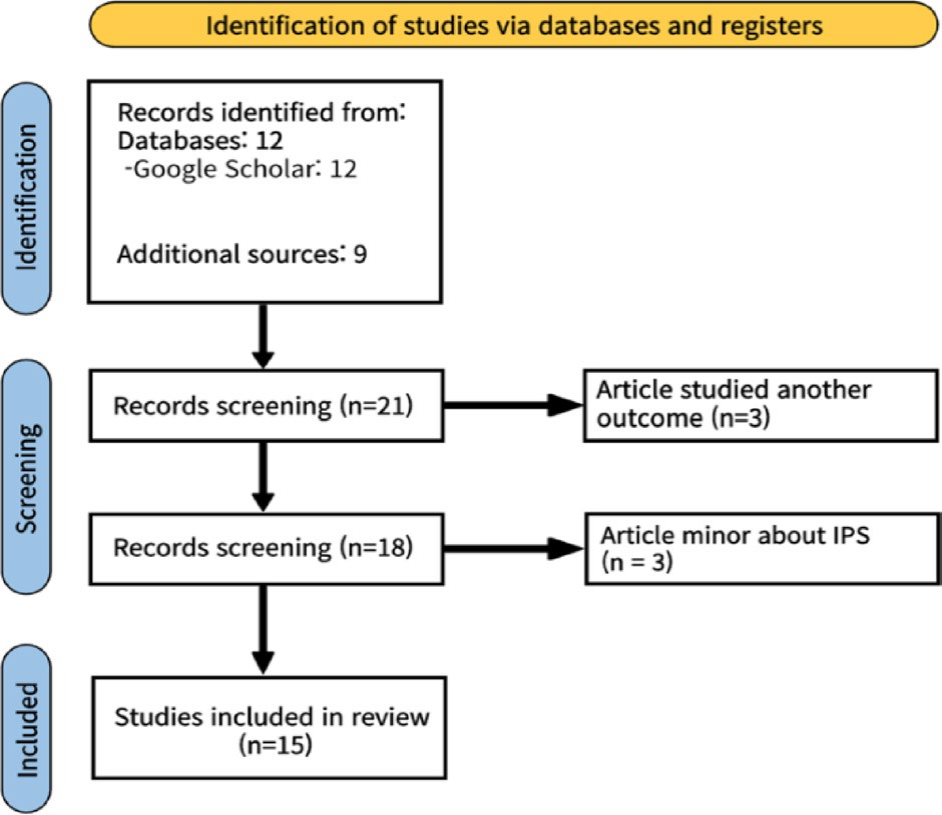
Studies screening process.

Through Google Scholar and Google Web Search, a total of 12 academic papers and 9 presentation materials or reports were initially identified. Among these, studies that, while related to IPS, were overly technical or focused primarily on ontologies were excluded, as they did not align with the objectives of this study. Likewise, documents with overly narrow scopes or those that did not primarily address IPS were also excluded. The exclusion process is illustrated in (Fig. 2).

As a result of the screening process, 9 academic papers, 3 presentation slides, and 3 reports were selected for inclusion (Table 3).

**Table 3:** Selected paper.

No	Ref	Publisher/Year	Goal	Challenges
1	The International Patient Summary: Proposal for a National Implementation for Cyprus^24^	Cyprus/2023	Development of a prototype IPS-based Electronic Health Record (EHR) and proposal for national implementation.	Only technical implementation and initial evaluation were conducted; it has not been broadly validated in real healthcare settings (public and private hospitals).
2	The International Patient Summary Standard and the Extensibility Requirement^25^	UK/2021	Analysis of the concept of the IPS summarization requirement and discussion on its sustainability.	The standard extension methods proposed are still in their early stages and require wide validation in large-scale medical environments.
3	The International Patient Summary and Validation in Common Use Cases in Japan^2^	Japan/2023	Discussion of IPS application and validation strategies in Japan.	Due to structural limitations of SS-MIX2 data, some items (medical history, devices, vaccinations) had to be manually entered from other sources. Unstructured text data (e.g., reason for admission, physical exam results, nursing summary, discharge status) were not fully represented, requiring additional approaches like natural language processing.
4	The Brazilian International Patient Summary Initiative^11^	Brazil/2024	Description of the IPS development process in Brazil and its contribution to the national digital health strategy.	Current RNDS data only includes information on COVID-19, Monkeypox tests, vaccinations, and some primary care data, which is insufficient for a comprehensive patient summary. Information on medical devices has not yet been implemented.
5	Exploring the Clinical Value of the International Patient Summary^26^	Netherlands/2024	Systematic review exploring the clinical value, barriers, and facilitators of IPS.	The study focused on general practitioners (GPs) in the Netherlands, limiting the generalizability of findings to other countries and healthcare settings. The limited experience with IPS in the study environment also affects result universality.
6	Patient Summaries: An International Perspective^27^	EU, US, Japan, Brazil/2026	Comparison of national patient summary strategies and emphasis on the need for international standardization.	As the workshop was the main method, there was no empirical data or statistical analysis. International comparisons were primarily qualitative.
7	IPS and the Summarization Requirement^28^	UK/2021	Examination of the difficulties in implementing IPS summarization and the need for standardization.	The current IPS standard only defines datasets and lacks detailed processes for summarization (e.g., how to select data). There is a need to develop detailed workflows and apply automation using AI.
8	International Patient Summary Standard Based on Archetype Concepts^29^	Bulgaria/2020	Implementation of IPS software based on archetype concepts and analysis of openEHR/EN ISO 13,606 application.	The IPS standard is in an early phase and has not yet been widely adopted or evaluated for user experience in real medical settings. Structural incompatibility between EN ISO 13,606 archetypes and openEHR makes direct conversion difficult.
9	eHealth4U: A DEMO of a Prototype National Electronic health Record for Cyprus^30^	Cyprus/2023	The goal of the paper is to present eHealth4U, a prototype national EHR system for Cyprus that complies with national legislation and international standards (HL7 FHIR, SNOMED CT, LOINC), aiming to support interoperability, the International Patient Summary, and user-friendly access for healthcare providers and citizens.	Key challenges include designing suitable FHIR profiles, ensuring usability through UX collaboration, maintaining compliance with GDPR and national privacy laws, and overcoming technical and organizational barriers to enable seamless health data exchange.
10	*The IPS Standard and the Extensibility Requirement^31^	CEN/TC 251, HL7 Italy, IHE /2020	Explanation of IPS extensibility requirements and various application scenarios.	As the IPS standard was in its early stage, issues of data extensibility were discussed, but validation for wide usability and applicability in real settings is still lacking. Governance challenges remain in achieving clinical consensus and updating reference models.
11	*G7 International Patient Summary Roadmap^32^	G7 Digital Health Group /2021	Roadmap for IPS implementation in G7 countries, definition of minimal dataset, and principles for patient data access.	Differences in health information management regulations and data protection laws across countries complicate seamless cross-border data transfer. The inconsistent use of terminologies like SNOMED CT also poses challenges for data consistency and interoperability.
12	*FprEN 17269:2019 - Health Informatics — The International Patient Summary^33^	CEN/TC 251 (European Committee for Standardization)/2019	Standard document defining the dataset, structure, and elements of the International Patient Summary.	The data elements defined in the IPS standard may not meet al.l the specific needs of complex clinical situations or specialties. Legal, administrative, and privacy issues vary by country, posing implementation challenges.
13	*The Value of the International Patient Summary in Canada^34^	Digital Health Canada, CHIEF Executive Forum /2021	Analysis of clinical and systemic effects of IPS adoption in Canada and proposal for implementation strategy.	Concerns about time and cost investment, as well as the immaturity of standardized data collection across Canada, lead to regional disparities. The workload of healthcare providers and the tendency to expect a perfect system from the outset may delay adoption.
14	*International Patient Summary Standards for Global Health^35^	HL7 Foundation, Trillium II /2019	Development of international IPS standards through the Trillium II project and presentation of diverse use cases.	Legal and administrative complexities due to differing national laws and data protection regulations hinder seamless system interoperability. Inconsistent use of common medical terminologies like SNOMED CT may lead to data consistency and interoperability issues.
15	*HISO 10099:2022 NZ International Patient Summary (NZIPS)^36^	New Zealand Ministry of Health /2022	Proposal of NZIPS standard adapted to the New Zealand environment, based on FHIR, and release of a public draft.	As a draft standard, large-scale implementation and practical use in clinical environments have yet to be validated.

*Gray paper.

### Step 1.2 literature review

A summary and analysis of the 17 selected documents revealed that IPS has emerged as a global initiative aimed at realizing healthcare data interoperability and patient-centered care. While implementation strategies and approaches vary across countries, IPS is being leveraged as a tool to enhance national healthcare systems and serves as a foundation for international collaboration. Notably, the “minimal dataset” defined by IPS—comprising Required and Recommended elements—is recognized as a standardized exchange unit with both practicality and scalability across diverse clinical contexts.

### Step 2 gap analysis between KR Core IG profiles and IPS IG profiles

#### Step 2.1 mapping of clinical and technical definitions between KR Core IG profiles and IPS IG profiles

Based on an analysis of the clinical meanings and technical definitions of the IPS Required and Recommended elements—namely, Allergy and Intolerance, Medication Summary, Problems, Immunizations, History of Procedures, Results, and Medical Devices—it was confirmed that IPS IG^3^ includes dedicated profiles encompassing each of these components.

A comparative mapping was then conducted between the IPS IG profiles and those defined in the KR Core IG profiles. As a result, seven IPS profiles—AllergyIntolerance (IPS), DiagnosticReport (IPS), Medication (IPS), MedicationRequest (IPS), Procedure (IPS), Immunization (IPS), and Condition (IPS)—were found to correspond to ten KR Core profiles: KR Core AllergyIntolerance Profile, KR Core DiagnosticReport for Diagnostic Imaging, KR Core DiagnosticReport for Function Tests, KR Core DiagnosticReport for Laboratory Results, KR Core DiagnosticReport for Pathology Results, KR Core Medication Profile, KR Core MedicationRequest Profile, KR Core Procedure Profile, KR Core Condition Profile for Chief Complaint, KR Core Immunization Profile, and KR Core Condition Profile for Encounter Diagnosis.

While some profiles demonstrated one-to-one mappings, several instances involved one IPS profile corresponding to multiple KR Core profiles, resulting in one-to-many mappings. Meanwhile, the IPS profiles MedicationStatement (IPS), Device (IPS), Device – Performer or Observer (IPS), and DeviceUseStatement (IPS) had no corresponding profiles in the KR Core IG (Table 4).

**Table 4:** Results of KR Core IG and IPS IG mapping.

	IPS IG	KR Core IG
IPS required	Allergyintolerance (IPS)	KR Core allergyintolerance profile
	Medication (IPS)	KR Core medication profile
	MedicationRequest (IPS)	KR Core medicationrequest profile
	Condition (IPS)	KR Core condition profile for chief complaint KR Core condition profile for encounter diagnosis
	MedicationStatement (IPS)	NO MATCH
IPS recommended	DiagnosticReport (IPS)	KR Core diagnosticreport profile for diagnostic imaging KR Core diagnosticreport profile for function tests KR Core diagnosticreport profile for laboratory results KR Core diagnosticreport profile for pathology results
	Procedure (IPS)	KR Core procedure profile
	Immunization (IPS)	KR Core immunization profile
	Device (IPS)	NO MATCH
	Device-performer or observer (IPS)	
	DeviceUseStatement (IPS)	

### Step 2.2 gap analysis of profile resource data between mapped KR Core and IPS IG profiles

Although the structural composition of the resources is largely similar, discrepancies were identified in the coding systems and categories used by certain profiles between IPS IG profiles and KR Core IG profiles. For example, while the DiagnosticReport profile in IPS IG employs LOINC codes, KR Core IG utilizes EDI codes. Similarly, the Procedure profile in IPS IG adopts SNOMED CT codes, whereas the corresponding KR Core profile uses EDI codes. Additionally, the Condition profile in KR Core is based on KCD codes, while IPS uses SNOMED CT codes for the same profile. In contrast, both the AllergyIntolerance and Medication profiles employ WHO ATC codes, demonstrating consistency between the two guides.

These differences indicate that in order to enable the KR Core to support IPS and broader international standards, adjustments or augmentations to EDI codes are essential. Since 2021, the Health Insurance Review and Assessment Service (HIRA) has publicly provided EDI–SNOMED CT mapping tables, starting with “Chapter 9: Treatment and Surgery Fees,” and expanding to include mappings for chapters on basic medical fees, laboratory tests, imaging and radiation therapy, medications, injections, anesthesia, physical therapy, and psychiatric therapy, comprising a total of 10,990 items^37^.

Furthermore, the Korean Health Information Standards (HINS) published a SNOMED CT mapping table for 14,402 KCD7 items, excluding traditional Korean medicine diagnoses^38^. These developments confirm the feasibility of mapping EDI and KCD codes—used in KR Core—to SNOMED CT. This suggests that if a system exists to convert KR Core data to international standards in advance, the data can be utilized in a globally interoperable manner.

### Step 3 design of a Korean IPS data pipeline utilizing FHIR data from the “My Health Record” application

#### Step 3.1 transformation of FHIR data from “My Health Record” app into IPS IG format

The data extracted from the My Health Record app included six data files related to medication records and immunizations from three individuals. In the case of Medical MyData from ‘SNUH’, data were obtained from a single individual. Among the three study participants, only one had retrievable medical records (e.g., surgery or consultation history) accessible through the MyData platform at this specific institution. All extracted data were provided in .json format, structured according to the FHIR standard.

The medication record data had a ResourceType of MedicationDispense. Analysis revealed the presence of Patient, Organization, and Medication resources, among which the Medication resource, required by IPS IG, was examined in detail. Unlike IPS IG specifications, this resource used a contained structure to hold additional data, and its system values were labeled as “undefined” or “resource identifier (undefined).” Moreover, redundant data were found across several resources.

The immunization data had a ResourceType of Immunization, which aligned with IPS IG requirements. However, similar to the medication records, this resource exhibited issues such as the use of contained elements and ambiguous system values.

The Medical MyData from ‘SNUH’ included resources such as MedicationRequest, Procedure, Patient, Organization, and Encounter. Among these, the MedicationRequest and Procedure resources were analyzed based on IPS IG requirements. However, the source data contained redundant nested resources—such as full Patient details embedded within other resources—and duplicate metadata that exceeded the scope of the IPS IG. In particular, the Procedure resource used EDI codes instead of the SNOMED CT codes mandated by the IPS IG, consistent with previous findings.

Based on these analyses, transformation prompts were developed for each raw dataset—medication record, immunization, and Medical MyData—to address identified discrepancies. Since validation is feasible only for individual resources, each prompt included a directive to extract or transform only one resource at a time. However, prompts designed for the final data pipeline allowed for the extraction and transformation of all relevant resources simultaneously.

Using ChatGPT-4o, the prompts were applied to each dataset (medication record, immunization, and clinical summary), and the final transformed resources—Medication, MedicationRequest, Procedure, and Immunization—were extracted. For the Procedure resource, a manual conversion was performed by the researcher using an EDI-to-SNOMED CT mapping table to meet IPS IG requirements.

The prompts used in this process are detailed in [Table 5].

**Table 5:** Generated prompt.

Resourcetype	Prompt
Immunization	You are a FHIR JSON data transformation expert specialized in International Patient Summary (IPS).
	Extract the first Immunization resource from the provided JSON and convert it into an IPS-compliant structure based on the following criteria:
	Objectives:
	Strictly conform to the HL7 FHIR IPS Immunization profile
	Flatten nested structures by removing contained resources
	Clean up metadata according to server requirements
	Transformation Rules:
	Preserve: resourceType, id, status, vaccineCode, occurrenceDateTime, lotNumber, primarySource
	Meta:
	Set profile to http://hl7.org/fhir/uv/ips/StructureDefinition/Immunization-uv-ips
	Remove versionId and lastUpdated (values will be assigned by the server)
	Set source to a https://myhealthway.go.kr. Do not use tag-like strings like “#SystemGenerated”
	Patient:
	Flatten the reference field
	Set reference value to “Patient/{id}” format
	Remove the nested resource object entirely
	Performer/Actor:
	Flatten references (e.g., generic actor or organization) to “Organization/{id}” or “Practitioner/{id}”
	Remove nested resource objects
	ProtocolApplied:
	Retain if present, ensuring targetDisease is preserved
	Cleanup:
	Remove encounter, location, note (unless critical)
	Remove any remaining nested resource objects
Medication	You are a FHIR JSON expert.
	The provided JSON may not conform to the standard FHIR Medication resource format. Modify the structure to match the FHIR Medication specification using the rules below.
	Objectives:
	Set resourceType to “Medication”
	Build a valid FHIR structure including ingredient, form, and text elements
	Ensure the final JSON is compatible with HAPI FHIR server
	Transformation Rules:
	identifier: retain the existing value, set a proper URL as system
	Remove Substance resources from contained
	Ingredient:
	Use itemCodeableConcept
	Include proper coding (code, system), strength (numerator with value & unit, denominator as form)
	Form: use SNOMED CT code to specify dosage form
	Text.div: must be in XHTML format with drug name and brief description
MedicationRequest	You are a FHIR JSON data transformation expert specialized in International Patient Summary (IPS).
	Extract the MedicationRequest resource from the provided JSON and convert it into an IPS-compliant structure based on the following criteria:
	Objectives:
	Strictly conform to the HL7 FHIR IPS MedicationRequest profile
	Flatten nested structures by removing contained resources
	Preserve clinical coding without altering original values
	Transformation Rules:
	Preserve: resourceType, id, status, intent, authoredOn
	Meta:
	Set profile to “http://hl7.org/fhir/uv/ips/StructureDefinition/MedicationRequest-uv-ips
	Medication Information:
	Retain medicationCodeableConcept object (coding and text)
	Do not convert to medicationReference
	Subject:
	Flatten the reference field
	Set reference value to “Patient/{id}” string format (extracting id from the nested resource)
	Remove the nested resource object entirely
	DosageInstruction:
	Retain the dosageInstruction array
	Preserve text, timing, route, and doseAndRate
	Keep the existing SNOMED CT coding for route
	Cleanup:
	Remove encounter, identifier, requester, reportedBoolean
	Remove any remaining nested resource objects
Procedure	You are a specialist in processing FHIR JSON-based data.
	Extract one Procedure resource from the provided JSON and simplify its structure based on the following criteria:
	Objectives:
	Retain only necessary fields
	Express code and subject references clearly
	Minimize metadata to essentials
	Transformation Rules:
	Retain: resourceType, id, meta, status, code, subject
	Meta: keep only versionId, lastUpdated, and source
	Code:
	Use only coding[0]
	If system is missing, set to “http://snomed.info/sct”
	Keep code and display
	Set text to the same value as code
	Subject: “reference”: “Patient/{id}” format using the existing reference to extract {id}

#### Step 3.2 verification of transformed FHIR data from the my Health record application

The validation results confirmed the effectiveness of the hybrid workflow, where human expertise defined the semantics while the AI handled the structural transformation.

First, the HAPI FHIR Server confirmed that the AI-generated JSON structure strictly adhered to the FHIR R4 base syntax without format errors, validating the model’s capability in syntactic generation (excluding expected errors regarding missing server-side references).

**Fig. 3:**
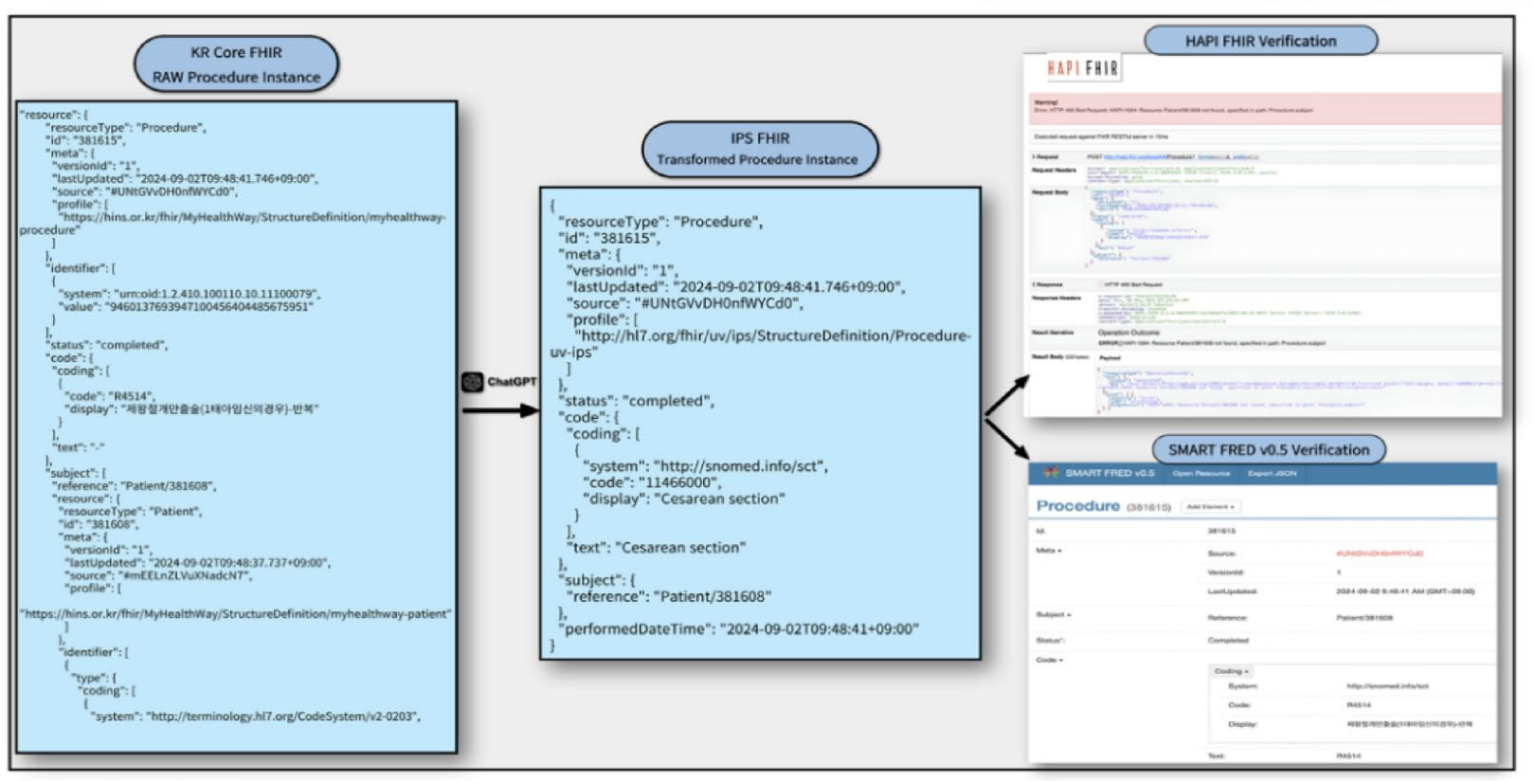
Example of prompt-based validation: procedure instance.

Second, semantic compliance with the IPS IG was ensured through manual replacement. As shown in [Figure 3], the Procedure instance was finalized by manually replacing the source EDI code with the valid SNOMED CT code (11466000) and populating mandatory elements (e.g., performedDateTime). This process ensured that the clinical codes were directly derived from the official mapping table rather than inferred by the AI. This human-verified instance was successfully recognized by SMART FRED, demonstrating that the final data artifacts fully satisfied IPS requirements.

#### Step 3.3 design of the data pipeline

**Fig. 4:**
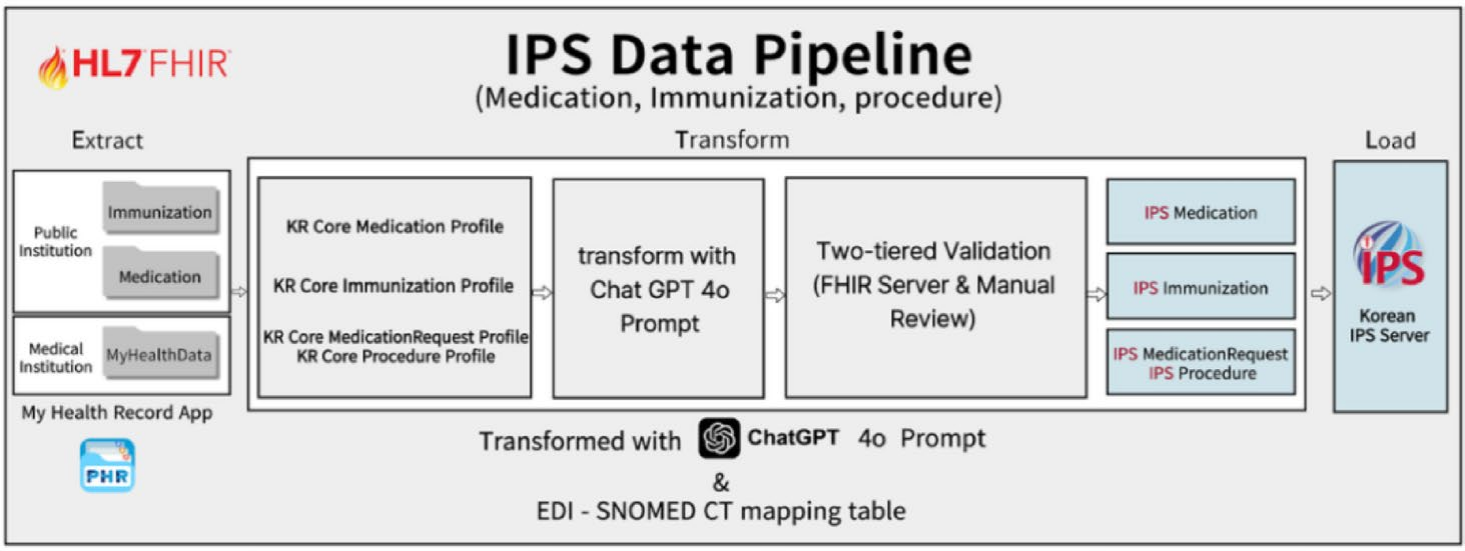
Designed IPS data pipeline.

A data pipeline was designed for the validated profiles: Medication, Immunization, Procedure, and MedicationRequest. The pipeline consists of a process in which the medication records, immunization data, and Medical MyData from “SNUH”, extracted via the My Health Record application, are transformed using four specifically designed prompts executed through ChatGPT-4o, verified for structural and semantic compliance via a two-tiered validation process, and subsequently stored as final, structured data (Fig. 4).

### Step 4 derivation of user requirements

#### Step 4.1 persona and scenario-based identification of user needs

At the initial stage of designing the Korean IPS system, brainstorming and mind mapping sessions were conducted. One participant, a PhD in health informatics and a nurse with several years of clinical experience, and another participant, a senior undergraduate student majoring in nursing, freely discussed the topic of “utilizing the Korean IPS.” Based on their input, a mind map was constructed. The PhD expert contributed perspectives grounded in clinical practice and informatics knowledge, while the undergraduate student offered insights combining academic and clinical training experiences. As a result, key themes such as “emergency situations,” “emergency departments,” and “patients with underlying conditions” were identified.

**Fig. 5:**
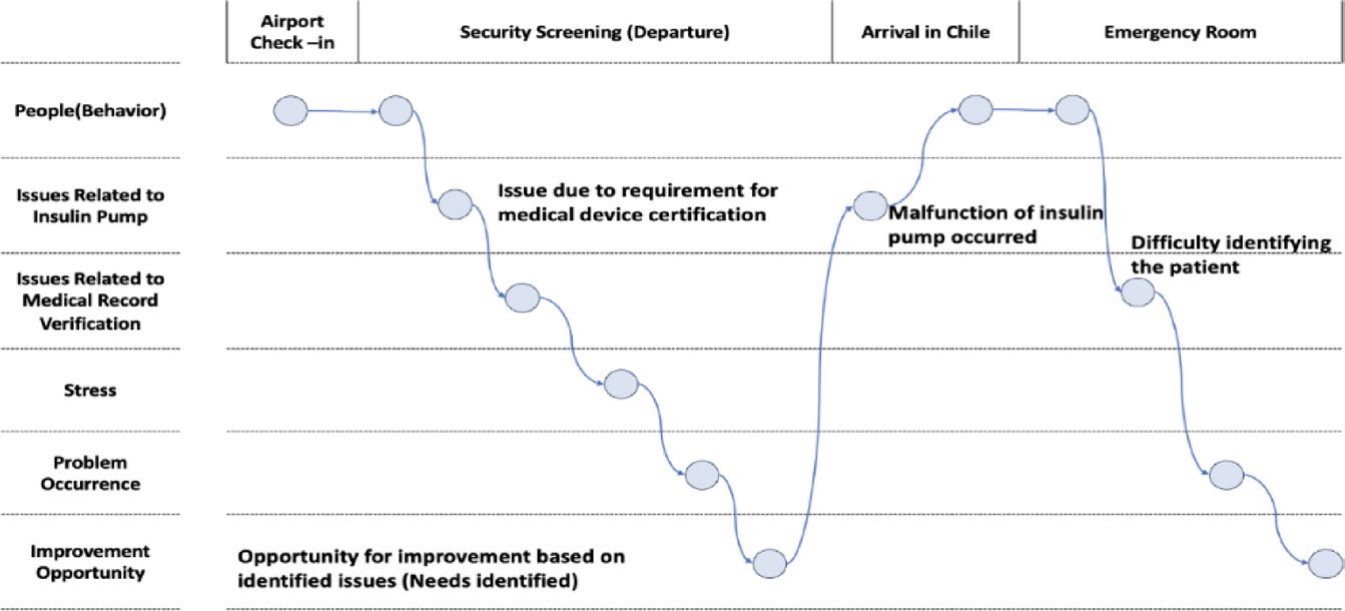
Persona A CJM.

**Fig. 6:**
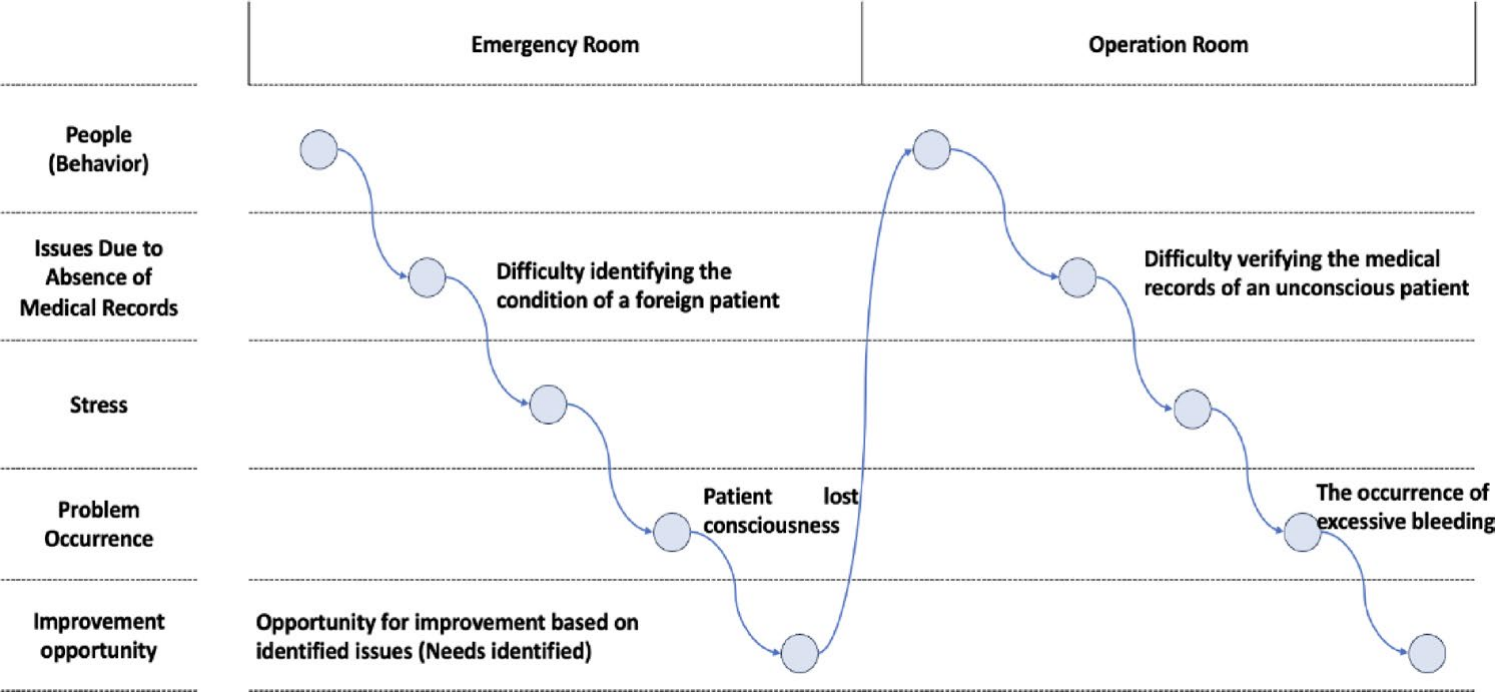
Persona B CJM.

Based on these findings, two personas were developed: “Persona A,” a patient with type 1 diabetes, and “Persona B,” an emergency medicine professor. These personas were derived from the dominant keywords identified during the mind mapping process, as well as data from the Health Insurance Review and Assessment Service (HIRA), which reported diabetes as the most prevalent chronic condition associated with emergency incidents in 2023, affecting approximately 4,068,318 individuals^26^. Scenarios were constructed for each persona, and corresponding Customer Journey Maps (CJMs) were created to visualize and analyze their interactions with the IPS system (Figs. 5 and 6).

Persona A – Patient with Type 1 Diabetes.

Name: Minjun Choi (28, Male).

Condition: Type 1 Diabetes mellitus.

Occupation: Software Developer.

Medical Device: Currently using an insulin pump.

Scenario:

Minjun Choi, a software developer with type 1 diabetes, traveled to Chile on a business trip. Unaware of the need for additional medical documentation when traveling internationally, he departed without proper preparation, wearing his insulin pump. During check-in, he discovered that the required documentation had been placed in checked luggage. As the insulin pump contains a needle, he was asked to verify that it was a medical device and to prove his diabetes diagnosis—something he was unable to do at that moment (A.1). After extensive negotiation, the situation was resolved through conversation with airport authorities.

Upon arrival in Chile, his insulin pump malfunctioned, rendering it unable to deliver insulin. Minjun attempted a manual injection, but due to unfamiliarity with dosage calculations, he administered an excessive amount of insulin. This resulted in a hypoglycemic episode. Although he managed to reach a nearby emergency room, local medical staff were unable to access sufficient medical history, which delayed appropriate care (A.2). Moreover, as Minjun lost consciousness, his colleagues struggled to communicate his condition to the healthcare team due to language barriers and a lack of medical knowledge (A.3). Prolonged hypoglycemia poses a significant risk of severe brain damage and central nervous system complications, underscoring the critical need for pre-prepared medical information and streamlined communication in emergency settings.

Persona B – Emergency Medicine Professor at a Tertiary Hospital.

Name: Minjeong Kim (38, Female).

Occupation: Professor of Emergency Medicine.

Scenario:

While on duty in the emergency department, Professor Minjeong Kim encountered Dmitry, a patient of Russian nationality who had been brought in via ambulance without a guardian. Due to language barriers, it was extremely difficult to obtain a medical history (B.1). As the patient’s condition deteriorated—marked by declining blood pressure and abdominal distension suggesting intra-abdominal bleeding—emergency surgery became imperative. A transfusion was needed due to massive hemorrhage, but the absence of blood type information necessitated the use of universal donor blood. Fortunately, an emergent blood test provided confirmation of the patient’s actual blood type.

However, the medical team was unaware that Dmitry had been taking aspirin for coronary artery disease, and this undetected information resulted in uncontrolled bleeding during the operation (B.2). Although the surgical team eventually managed to stabilize the patient after a prolonged operation, the situation could have led to fatal consequences. This case highlights the urgent need for accessible, accurate patient information in cross-border and emergency medical contexts.

The following user requirements were identified through the personas:

*A.1 – A system that provides information about medical devices*.

*A.2*,* B.2 – A system that allows rapid access to medical information*.

*A.3*,* B.1 – A system that overcomes language barriers*.

*B.2 – A system accessible even when the patient is unconscious*.

These scenarios and derived needs were validated by four experts: one representative from a diabetes patient advocacy group (Smart Patient), two medical informatics specialists, and one emergency medicine specialist with expertise in medical informatics. All experts affirmed the clinical relevance and practical necessity of the proposed use cases and requirements.

### Step 4.2 expert consultation-based identification of user needs

Expert feedback was obtained through four structured questions, yielding the following insights:

#### Q1

What is the most effective way to display IPS documents in clinical settings?

Most experts favored a summary view as the default display format for IPS documents. They emphasized that when integrating IPS into existing EMR systems, the IPS content should be visually separated to prevent information confusion. Some respondents also suggested offering a timeline-based view as an optional feature. For example, one emergency physician noted, “A summary view integrated into the EMR screen would be ideal for clinicians,” while a UX designer proposed, “A summary card within the EMR, with an option to switch to a timeline view, would enhance usability.” These comments imply that the IPS viewing interface should be summary-oriented by default, yet flexible enough to accommodate clinical context through alternative visualizations.

#### Q2

Which data elements should be prioritized during the initial pilot of the Korean IPS?

Experts agreed that allergies, medication history, and current medications should be prioritized. Some added that diagnostic reports, even without original images, could still be clinically useful if interpretation notes are included. A health data expert noted, “With current technology, allergies, medication history, recent test results, immunizations, and current medications are feasible. However, historical diagnoses and past medical history would require long-term development and validation.” A UX designer further suggested grouping essential emergency information to support scenario-based information structuring in the future.

#### Q3

What are your thoughts on a phased implementation starting with a minimal dataset?

All experts supported a phased approach beginning with a minimal dataset. One emergency physician remarked that certain IPS required elements may be technically difficult to implement and advocated a minimum viable product strategy with prioritized deployment. The UX designer emphasized maintaining the highest visual hierarchy for essential information, even as the dataset expands. These insights stress that clarity in information delivery and support for clinical decision-making should take precedence over data volume.

#### Q4

What are the critical requirements for ensuring the clinical reliability of IPS?

Experts consistently identified three core factors: clear indication of data sources, inclusion of up-to-date information, and the use of internationally standardized coding systems. A FHIR expert specifically stated that source identifiers should be detailed at the system level—e.g., “National Health Insurance Service,” “Seoul National University Hospital.” A UX designer recommended incorporating brand elements such as official certification logos, institutional signatures, and design consistency to enhance perceived trust. This reflects the view that the IPS should be recognized not just as a data bundle, but as a credible clinical document.

Expert-Derived User Requirements Summary:

UI: Summary view as default; visually separated from the EMR; timeline view as an optional user-triggered display.

Data: Begin with Required and Recommended elements; prioritize allergies and medication history; interpretation reports can substitute for original diagnostic images.

Reliability: Clear indication of source institutions; branding elements to enhance document credibility.

### Step 5 UI design

**Fig. 7:**
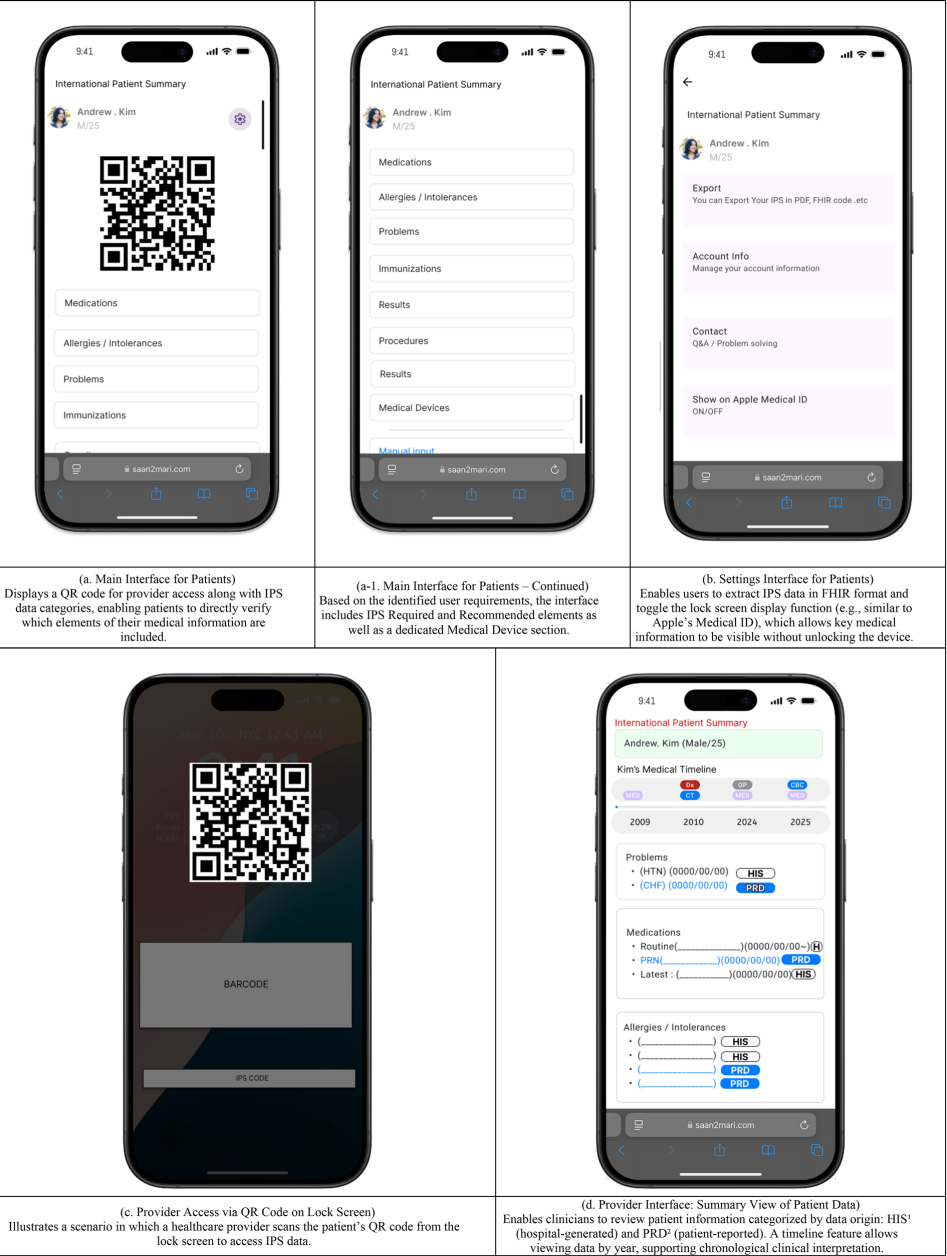

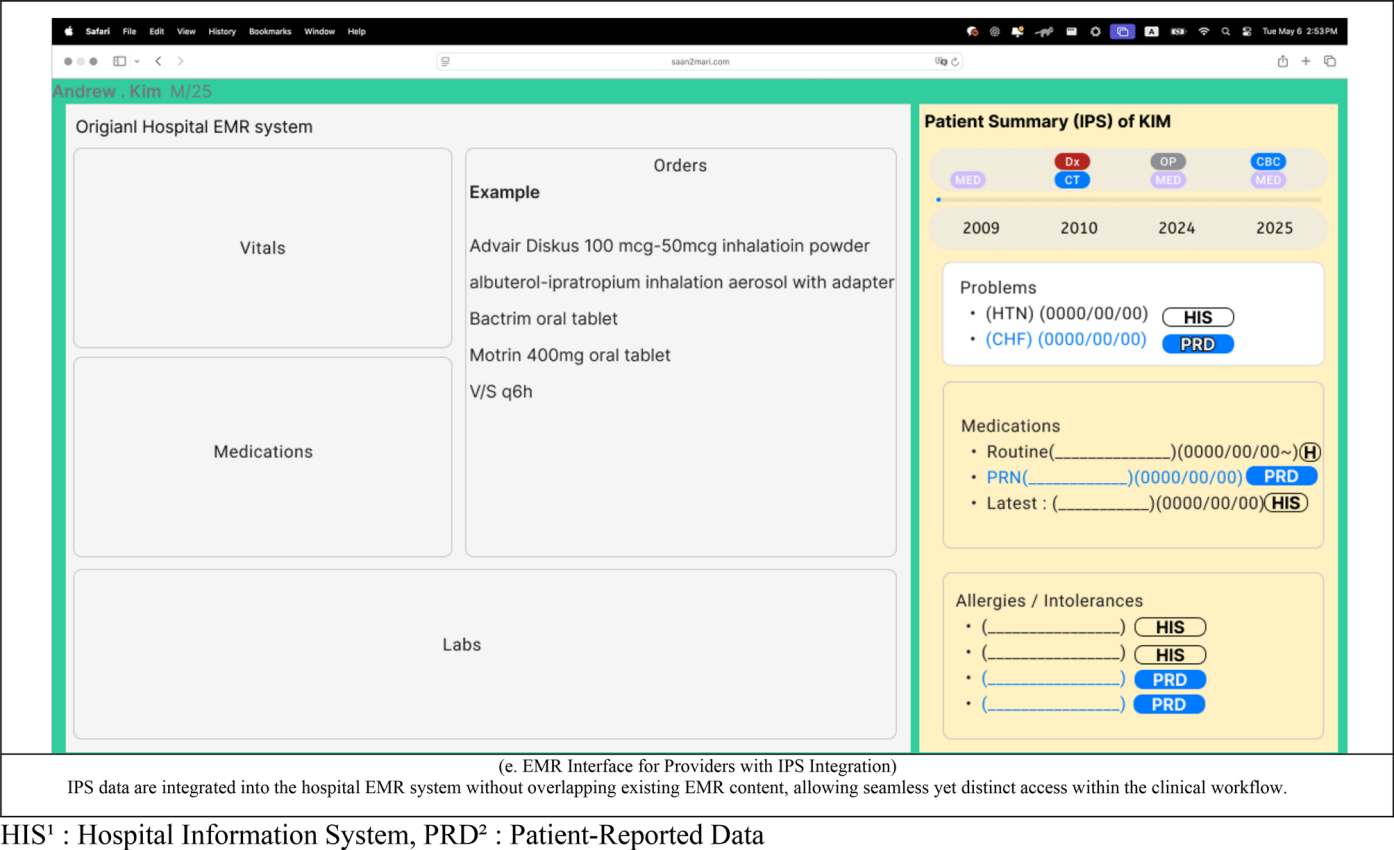
Designed UI. HIS^1^ : Hospital Information System, PRD^2^ : patient-reported data.

Given that countries such as the United States and Japan have implemented IPS through web-based applications, and considering the growing ubiquity of web-based electronic health record (EHR) systems, the IPS interface was designed as a web application. In addition, based on the previously derived user requirements, an EMR-integrated IPS interface was also designed to accommodate clinical workflows (Fig. 7).

## Discussion

This study systematically demonstrated the feasibility of implementing a Korean version of the IPS through four key phases: (1) gap analysis between KR Core IG and IPS IG, (2) transformation and validation of FHIR data extracted from the My Health Record application, (3) design of a data pipeline, and (4) user-centered UI development.

First, the literature review confirmed that IPS has been internationally recognized as a core standard for enabling health data interoperability and advancing patient-centered care^27,35^. The minimal dataset defined by IPS proved effective in enhancing data exchange and accessibility across various clinical contexts, thereby improving patient safety and continuity of care^11,33^. Furthermore, national case studies revealed diverse strategies and implementation models that offer meaningful insights for Korea’s adoption process^2,11,24,34^, highlighting the need for a customized strategy aligned with domestic healthcare policies and infrastructure^27,29^.

Second, the gap analysis revealed that 6 of the 7 IPS Required/Recommended profiles (AllergyIntolerance, Conditions, Medication, MedicationRequest, Immunization, DiagnosticReport, Procedure) could be mapped to KR Core profiles. However, profiles such as MedicationStatement and Device were not represented in KR Core, resulting in both 1:n mappings and unmapped areas. Additionally, discrepancies in coding systems (e.g., SNOMED CT, LOINC, EDI, KCD) were observed. While some prior studies addressed such issues by defining custom code systems, they were limited by small datasets^20^. These findings suggest that although KR Core is largely prepared to support IPS profiles, further research is needed to establish comprehensive mapping tables for converting domestic to international standards.

Third, the experimental data pipeline demonstrated the potential of using a hybrid approach—combining ChatGPT-4o for structural transformation with human expertise for semantic accuracy—to resolve inconsistencies in FHIR data. Validation was conducted using a two-tiered strategy: while the HAPI FHIR Server confirmed syntactic conformity to the FHIR R4 base structure, semantic alignment with IPS profiles was ensured through manual curation and visual verification via SMART FRED.

Notably, the manual replacement of local EDI codes with SNOMED CT codes, based on official mapping tables, enabled true international standardization of Procedure resources. This provides empirical support that, with official mapping tables and automated APIs, domestic EHR data can be rapidly converted into IPS-compatible formats. Prior studies also acknowledged the necessity of manual transformation and proposed NLP-based automation as future work^20^. In contrast, this study utilized generative AI to streamline the structural conversion process, while integrating human-in-the-loop verification to address the limitation of incomplete or missing codes.

Fourth, unlike previous studies that relied solely on persona-based scenarios^22,23^, this study incorporated practical insights from implementation experiences through expert consultation. Key requirements were identified, including rapid access to emergency information, prioritization of allergy and medication history, visual separation from existing EMRs, and support for summary and timeline dual views. These insights were reflected in the UI design for both mobile and EMR interfaces. The consensus among experts on the “minimal dataset first, gradual expansion” approach, and the recommendation to adopt a summary card plus timeline UI structure for the Korean IPS, reinforce the clinical relevance and usability of the proposed design. Future studies should include large-scale usability testing and multilingual accessibility evaluation to ensure clinical acceptance.

The key contributions of this study include: (1) identifying global IPS trends and limitations through a literature review, (2) demonstrating that KR Core IG can fulfill most IPS Required/Recommended elements, (3) constructing an IPS-compatible FHIR data pipeline using personal health data from the "My Health Record" application, and (4) developing a Korean IPS UI that integrates clinical, industrial, and UX perspectives. These outcomes could serve as critical infrastructure for patient safety in cross-border care, disaster response, and telemedicine, in synergy with initiatives such as the Health Information Highway, next-generation EMR projects, and national MyData services.

However, this study is constrained by several limitations.

First, regarding data availability, the sample size was limited to a small cohort from a single institution. Specifically, the SNUH dataset was restricted to a single individual because, among the three participants, only one had retrievable medical records (e.g., surgery or consultation history) accessible via the MyData platform; the others lacked visit records or faced integration limitations. However, since the primary objective was to verify the technical feasibility of the transformation pipeline rather than to conduct a statistical analysis, this sample size was deemed sufficient.

Second, regarding standardization, the analysis excludes the MedicationStatement and Device profiles due to their absence in the current KR Core. Additionally, a complete mapping table between KCD and LOINC is currently unavailable, representing a semantic limitation of the present system.

Third, regarding the methodology, the study faces challenges related to the inherent non-deterministic nature of generative AI outcomes, which may affect consistency. Furthermore, the manual verification process required for terminology mapping imposes scalability constraints for large-scale implementation.”

Future work should address these limitations by developing a fully automated terminology mapping module to replace manual intervention; implementing a continuous validation framework (e.g., rule-based logic or SHACL) to guarantee the reliability of non-deterministic AI outputs in production environments; refining automated mapping algorithms using large-scale, multi-institutional FHIR datasets; and establishing certification frameworks for IPS reliability.

In summary, this study demonstrates that a four-phase approach—literature review, gap analysis, data pipeline implementation, and user-centered UI design—can serve as a practical foundation for realizing a Korean IPS. With continued research and policy support, Korean healthcare can join the global interoperability movement and accelerate the transition to patient-centered digital health.

## Data Availability

The datasets used during the current study are available from the corresponding author on reasonable request.

## Declarations

### Figure Right

All of the figures were taken by first author Byeonggu Kim.

### Used Software Information

We used ChatGPT-4o(Ver. 1.2025.175). Software link is https://openai.com.
